# Identification of natural killer markers associated with fatal outcome in COVID-19 patients

**DOI:** 10.3389/fcimb.2023.1165756

**Published:** 2023-06-05

**Authors:** Nadine Tarantino, Elena Litvinova, Assia Samri, Cathia Soulié, Véronique Morin, Alice Rousseau, Karim Dorgham, Christophe Parizot, Olivia Bonduelle, Alexandra Beurton, Makoto Miyara, Pascale Ghillani, Julien Mayaux, Raphael Lhote, Jean-Marc Lacorte, Anne-Geneviève Marcelin, Zahir Amoura, Charles-Edouard Luyt, Guy Gorochov, Amélie Guihot, Vincent Vieillard

**Affiliations:** ^1^ Sorbonne Université, Inserm, CNRS, Centre d’Immunologie et des Maladies Infectieuses (CIMI-Paris), Hôpital Pitié-Salpêtrière, Paris, France; ^2^ Assistance Publique-Hôpitaux de Paris (AP-HP), Hôpital Pitié-Salpêtrière, Département d’Immunologie, Paris, France; ^3^ Sorbonne Université, Inserm, Institut Pierre Louis d’Epidémiologie et de Santé Publique (iPLESP), Assistance Publique – Hôpitaux de Paris (AP-HP), Hôpital Pitié-Salpêtrière, Laboratoire de Virologie, Paris, France; ^4^ Assistance Publique-Hôpitaux de Paris (AP-HP), Hôpital Pitié-Salpêtrière, Service de Médecine Intensive-Réanimation et Pneumologie, Paris, France; ^5^ Sorbonne Université, Inserm UMRS Neurophysiologie Respiratoire Expérimentale et Clinique, Assistance Publique – Hôpitaux de Paris (AP-HP), Paris, France; ^6^ Service de Médecine Interne 2, Institut E3M, Assistance Publique – Hôpitaux de Paris (AP-HP), Hôpital Pitié-Salpêtrière, Paris, France; ^7^ Sorbonne Université, Inserm, UMRS1166-ICAN Institute of Cardiometabolism and Nutrition, Paris, France; ^8^ Service de Biochimie Endocrinienne et Oncologique, Assistance Publique – Hôpitaux de Paris (AP-HP), Hôpital Pitié-Salpêtrière, Paris, France

**Keywords:** fatal outcome, COVID-19, natural killer (Nk) cell, SARS-CoV-2 infection, tNF-alpha

## Abstract

**Introduction:**

Increasing evidence has shown that coronavirus disease 19 (COVID-19) severity is driven by a dysregulated immunological response. Previous studies have demonstrated that natural killer (NK) cell dysfunction underpins severe illness in COVID-19 patients, but have lacked an in-depth analysis of NK cell markers as a driver of death in the most critically ill patients.

**Methods:**

We enrolled 50 non-vaccinated hospitalized patients infected with the initial virus or the alpha variant of SARS-CoV-2 with moderate or severe illness, to evaluate phenotypic and functional features of NK cells.

**Results:**

Here, we show that, consistent with previous studies, evolution NK cells from COVID-19 patients are more activated, with the decreased activation of natural cytotoxicity receptors and impaired cytotoxicity and IFN-γ production, in association with disease regardless of the SARS-CoV-2 strain. Fatality was observed in 6 of 17 patients with severe disease; NK cells from all of these patients displayed a peculiar phenotype of an activated memory-like phenotype associated with massive TNF-α production.

**Discussion:**

These data suggest that fatal COVID-19 infection is driven by an uncoordinated inflammatory response in part mediated by a specific subset of activated NK cells.

## Introduction

Multi-system injuries and death observed in patients infected by severe acute respiratory syndrome coronavirus 2 (SARS-CoV-2) are thought to be associated with immune dysfunctions (Guihot et al., 2020; Kuri-Cervantes et al., 2020; Mathew et al., 2020; McKechnie and Blish, 2020). However, the exact mechanisms leading to severe forms of coronavirus disease 19 (COVID-19) are still largely unknown, in particular with regard to whether the cellular immune responses are protective or if, on the contrary, this leads to immunopathological mechanisms resulting in lung injury. Several studies performed in patients requiring admission to the intensive care unit (ICU) have reported increased antiviral responses mediated by lymphocyte T cells and mucosal-associated invariant T (MAIT) cells, induced by uncoordinated inflammatory responses (Peng et al., 2020; Schub et al., 2020; Youngs et al., 2021; Guihot et al., 2022). However, the lack of strong associations between conventional spike-specific T cell functions and neutralizing antibody titers with a fatal outcome suggests that neither excessive nor defective antigen-specific responses contribute to mortality in critical COVID-19 infection; this raises questions about the role of other immune components and more particularly of natural killer (NK) cells, which play a key role in response to acute viral infections.

NK cells are critical elements of innate immunity which are capable of implementing cytotoxic and immunomodulatory responses. Such functions are under the control of a finely-tuned equilibrium between activating and inhibitory receptors. In the steady state, NK cells predominantly express inhibitory receptors, such as killer cell immunoglobulin-like receptors (KIRs) and NKG2A, which recognized HLA class-I molecules. After infection, the engagement of activating receptors can counterbalance inhibitory receptor signals, allowing NK cells to mount specific cytotoxic and immunoregulatory responses. These receptors include NKG2D, the natural cytotoxic receptors NKp46 and NKp30, and NKG2C, whose expression is associated with certain viral infections Gasser and Raulet, 2006; Lopez-Verges et al., 2011; Sun and Lanier, 2011; Vivier et al., 2011). In COVID-19 patients, a decrease in peripheral NK cells was consistently reported (Guihot et al., 2020; Manickam et al., 2020; Maucourant et al., 2020; Wilk et al., 2020; Di Vito et al., 2022), suggesting the possible sequestration of NK cells in the lungs, which could either help to fight the virus or exacerbate damage in the lung tissues and consequently induce epithelial cell death (Liao et al., 2020; McKechnie and Blish, 2020). Functionally, NK cells exhibit reduced cytotoxicity in COVID-19, previously explained by an over-expression of the inhibitory NKG2A receptor (Zheng et al., 2020), but not confirmed by other groups. In fact, depending on the studies, other markers were found to be responsible, such as NKG2D, DNAM-1 or NKp30 (Varchetta et al., 2020), or even the presence of adaptive NKG2C^+^ NK cells which correlate with disease severity (Maucourant et al., 2020). Alternatively, it was also reported that granzyme A-expressing NK cells were negatively correlated with serum IL-6 levels in COVID-19 patients, suggesting a link to inflammation (20 Mazzoni et al., 2020).

In summary, these inconsistent data on NK cells further emphasize the need for new studies to better understand their role in SARS-CoV-2 infection and more specifically in patients who develop severe/critical forms of the disease. Therefore, in this study, we sought to specifically investigate the association of NK cells with severity of disease to better determine the potential role of NK-cell responses in contributing to or protecting from fatality in COVID-19 patients.

## Materials and methods

### Ethics statement

The protocol was approved by the ethics committee of Sorbonne Université for patients infected with the original strain who presented moderate and severe forms of COVID-19 (no. CER-2020-21 and CER-2020-31, respectively) and for patients infected by the Alpha variant (no. CER-2021-044). The patients provided written informed consent to participate in this study.

### Study participants

Fifty non-vaccinated patients infected by the original (WT) strain or Alpha variant (α) of SARS-CoV-2 admitted to the Pitié-Salpêtrière Hospital, Paris (France) were recruited to the study. Hospitalized patients were classified as having moderate (n=33) or severe (n=17) COVID-19 disease when treated in an intensive care unit requiring either non-invasive or mechanical ventilation. Detailed clinical parameters of the infected patients are shown in **Table 1**. The characteristics of patients included age, sex, co-morbidities and gravity scores like the Sequential Organ Failure Assessment (SOFA) score and/or the *Simplify Gravity Index (IGS II).* Peripheral blood samples of 25 healthy individuals obtained between 2008-2009 from the French Blood Bank (EFS; Etablissement Français du Sang) were used as negative controls.

**Table 1:** Demographic and clinical characteristics of patients admitted to hospital with COVID-19.

	Severe patients	Moderate patients	Moderate patients	P*
SARS-Cov-2 variant	WT	WT	Alpha	
Number (n)	17	18	15	
Sex; n of Female (%)	4 (23.5)	9 (50.0)	3 (20.0)	ns
Mean age in Yrs (ranged)	57 (23-71)	63 (27-94)	57 (31-82)	ns
Days post-symptom; mean ± SD	9 ± 5	11 ± 5	14 ± 6	ns
Co-morbidities				
Obesity; n (%)	11 (64.7)	4 (22.2)	6 (40.0)	0.0176
Diabetes (type 1 and 2); n (%)	7 (41.2)	4 (22.2)	3 (20.0)	ns
Chronic cardiac disease; n (%)	2 (11.8)	2 (11.1)	3 (20.0)	ns
Hypertension; n (%)	8 (47.1)	12 (70.6)	9 (60.0)	ns
Gravity scores				
IGS II at baseline; mean ± SD	41.6 ± 16.1	27.6 ± 12.4	ND	0.0099
SOFA; mean ± SD	11 ± 5	ND	ND	ND
Respiratory support (ECMO); n (%)	6 (35.3)	0 (0)	0 (0)	0.0076
ARDS; n (%)	7 (41.2)	1 (5.5)	0 (0)	0.0014
Mortality at hospital; n (%)	6 (35.3)	0 (0)	0 (0)	0.0076

WT, wild-type; ND, non-determined; ns, no significant; IGS II, Simplify Gravity Index; SOFA, Sequential Organ Failure Assessment; ECMO, Extracorporeal Membrane Oxygenation; ARDS, Acute respiratory distress syndrome; * statistical analyze: Fisher t tests, excepted for Age and Gravity scores (Mann-Whitney test) were performed between patients infected with WT SARs-CoV-2 strain with severe and moderate COVID-19.

### Diagnosis of SARS-CoV-2 viral variants

The diagnosis of COVID-19 was confirmed in all patients by SARS-CoV-2 carriage in the nasopharyngeal swab, as measured by real-time reverse transcription PCR (RT-PCR) analysis. RT-PCR SARS-CoV-2 positive samples were next screened to assess SARS-CoV-2 viral lineages with the TaqPath™ COVID-19 RT-PCR (ThermoFisher, Waltham, USA) and the VirSNiP SARS-CoV-2 Spike E484K (TIB Molbiol, Berlin, Germany), by the virologic department of the Ptité-Salpêtrière hospital (Teyssou et al., 2021).

### Flow cytometry analyses

To analyze CD3^-^CD56^+^ NK cells, frozen peripheral blood mononuclear cells (PBMCs) were used and stained with an appropriate cocktail of monoclonal antibodies (mAbs), described in the **Supplementary Table S3**, according to flow cytometry guidelines (Cossarizza et al., 2019). At least 20,000 CD45^+^ cells were acquired (Gallios, Beckman-Coulter) and then analyzed with FlowJo software version 10 (TreeStar), as previously described (Carbonnel et al., 2022). The gating strategy is shown in the **Supplementary Figure 1**. Furthermore, a hierarchical clustering analysis from NK markers whose expression is modulated during COVID-19 was carried out using the Genesis program (www.genome.tugraz.at), as described (Vieillard et al., 2010).

### NK-cell degranulation assays and cellular production of cytokines

NK cells were pre-treated overnight in the presence of interleukin (IL)-12 (10 ng/ml) plus IL-18 (100 ng/ml) to measure the intracellular production of IFN-γ and TNF-α by NK cells, or incubated with the standard HLA class-1 negative K562 target cells (ATCC CCL243), at an effector:target (E:T) cell ratio of 1:1, to measure degranulation in the presence of anti-CD107a mAb (#H4A3; Becton Dickinson) (**Supplementary Table 1**). Cells were thereafter incubated for 5 hours in the presence of Golgi Stop and Golgi Plug solutions (BD Biosciences) and then stained with NK cell-surface markers. Next, NK cells were fixed, permeabilized with a cytofix/cytoperm kit (Becton Dickinson) and then intracellularly stained with anti-IFN-γ and anti-TNF-α mAbs (**Supplementary Table 1**), as previously described (Carbonnel et al., 2022). At least 1000 CD3^-^CD56^+^ NK cells were acquired on a Gallios flow cytometer (Beckman Coulter) and then analyzed with Flow Jo version 10 (TreeStar, state).

### Statistical analyses

Statistical analyses were performed with Prism 8.0 software (GraphPad, CA, USA). The quantitative data are described as median values. The non-parametric Kruskal-Wallis test and a Dunn’s multiple comparison test were used for comparisons between groups. Correlations between variables were calculated using the non-parametric Spearman rank-order test. *P* values >0.05 were considered insignificant. Principal component analysis was used to graphically assess the separation between healthy donors and all groups of COVID-19 patients with regards to phenotypic and functional parameters, using XLSTAT.

## Results

### Demographic and clinical features of COVID-19 patients

A total of fifty infected patients were enrolled in our study. The demographic and clinical characteristics of the study population are shown in **Table 1**. No differences in demographic and clinical characteristics were found between the study groups of patients with moderate disease infected with the original strain (WT-M, n=18) or the Alpha variant (α-M, n=15). In contrast, patients with severe disease (WT-S, n=17) presented significantly increased gravity scores and ECMO and ARDS scores compared to patients with moderate COVID-19. Furthermore, among the 17 patients with severe disease, six (35%) died in hospital; they all presented co-morbidity factors and pulmonary infections (**Supplementary Tables 2, 3**).

### Phenotypic repertoire of NK cells from COVID-19 patients

**Figure 1:**
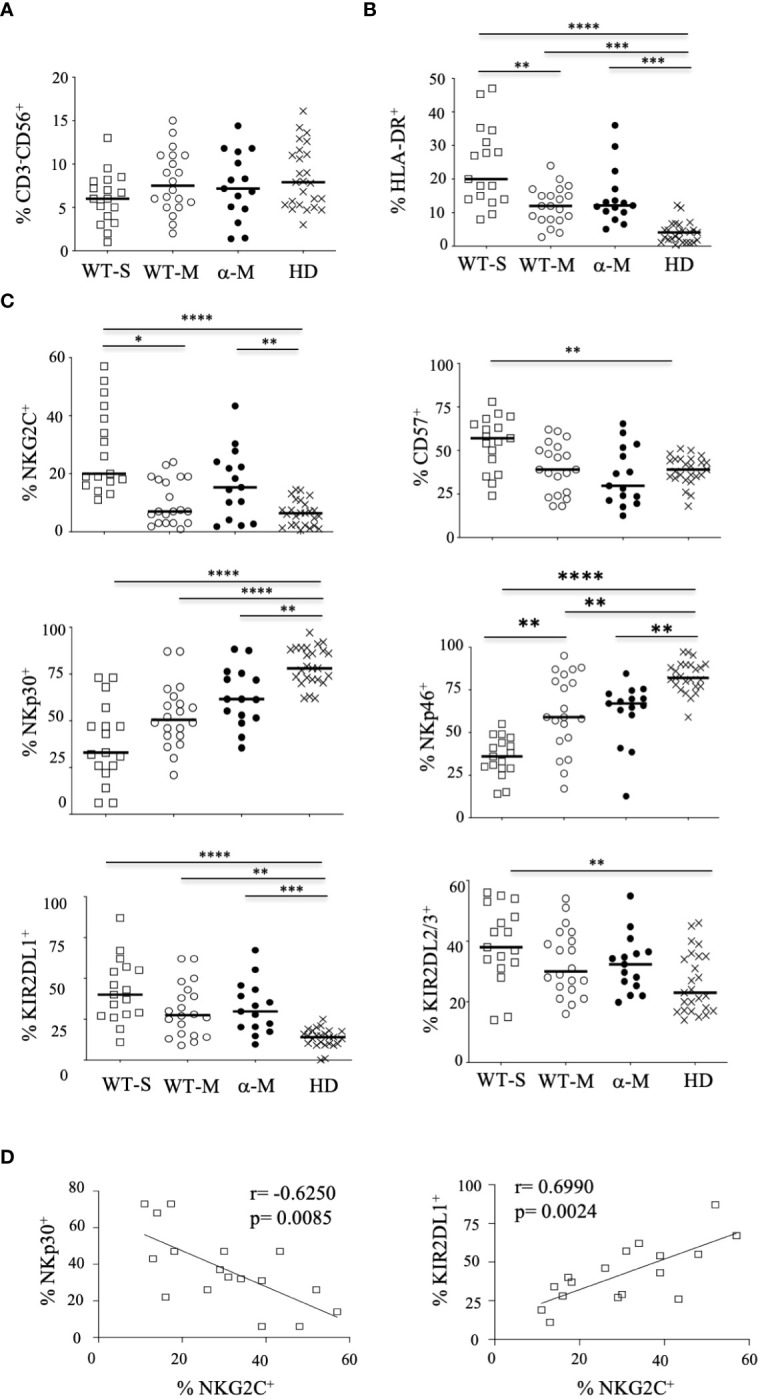
Phenotypic characteristics of NK cells from COVID-19 patients. **(A)** Percentage of CD3^-^CD56^+^ NK cells within the lymphocyte gate. **(B)** Percentage of HLA-DR^+^ activated cells among CD3^-^CD56^+^ NK cells. **(C)** Percentage of cells expressing cell-surface markers among CD3^-^CD56^+^ NK cells. Data are shown at the first time point (Pt1) for healthy donors (HD, n=25), patients with severe (WT-S, n=17) or moderate COVID-19 (WT-M, n=18) infected with the WT virus, or patients with moderate COVID-19 infected with the Alpha variant (α-M, n=15). Black lines represent the median. *p<0.05; **p<0.001; ***p<0.0005, ****p<0.0001. **(D)** Correlation between NKp30 or KIR2DL1 and NKG2C in NK cells from WT-S COVID-19 patients.

**Figure 2:**
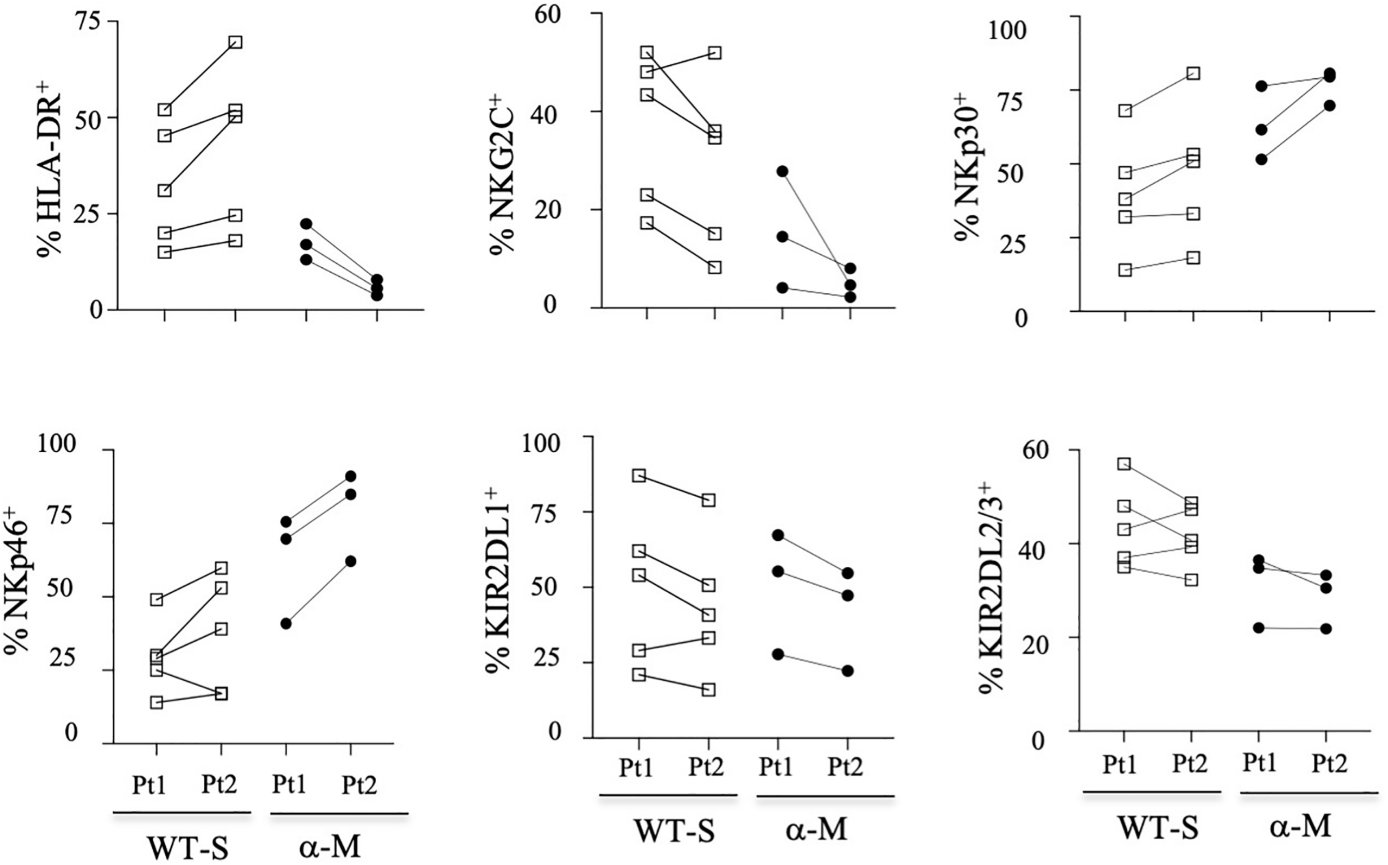
Kinetic study of NK-cell markers from COVID-19 patients. Data from some patients are shown at the two-time point (Pt1 and Pt 2) for healthy donors (HD), patients with severe (WT-S) or moderate COVID-19 (WT-M) infected with the WT virus, or patients with moderate COVID-19 infected with the Alpha variant (α-M). Black lines represent the median.

The overall proportion of CD3^-^CD56^+^ NK cells was comparable in COVID-19 patients with moderate or severe clinical forms and healthy donors (**Figure 1A**). Furthermore, the frequency of the CD56^bright^ subset was similar in samples from COVID-19 patients and healthy donors (**Supplementary Figure 2**). To better characterize these cells, we performed an extensive analysis of their cell-surface receptors early after inclusion (Pt1=Day 1 to 3); NK cells from COVID-19 patients were indistinguishable from those of the healthy donors concerning the following NK receptors: NKG2A, ILT-2 and KIR3DL1 (**Supplementary Figure 3**). Furthermore, we did not observe any significant phenotypic differences after infection with the WT virus or Alpha variant in patients who develop moderate clinical features. However, the frequency of NK cells expressing the cell-activation marker HLA-DR was increased in COVID-19 patients compared to healthy donors and even more significantly in patients with severe COVID-19 (p<0.0001) (**Figure 1B**). Furthermore, NK cells from COVID-19 patients over-express inhibitory KIR2DL1 or KIR2DL2/DL3 receptors, which specifically recognize HLA-C molecules, compared to healthy donors (**Figure 1C**). In the compartment of activating NK cell receptors, NKp30 and NKp46 are dramatically decreased in COVID-19 patients (p<0.0001) compared to healthy donors (**Figure 1C**). On the other hand, the key NKG2C marker of adaptive NK cells associated with viral infections (Stary and Stary, 2020) was significantly increased in COVID-19 patients compared to heathy donors (p<0.0001) and more specifically in patients with severe COVID-19 (**Figure 1C**). Moreover, in patients with severe COVID-19, NKG2C expression was negatively correlated with NKp30 (p=0.0085, r=-0.6250) and positively correlated with KIR2DL1 (p=0.0024, r=0.6990) (**Figure 1D**). Of note, by comparing the expression of markers at the two time points post-inclusion (Pt1=Day 1 to 3 and Pt2 =day 14 to 24), the frequency of markers remains abnormal at Pt2 in most patients with severe COVID-19, whereas most markers revert to healthy donor at Pt2 for those with moderate disease (**Figure 2**; **Supplementary Figure 3**).

**Figure 3:**
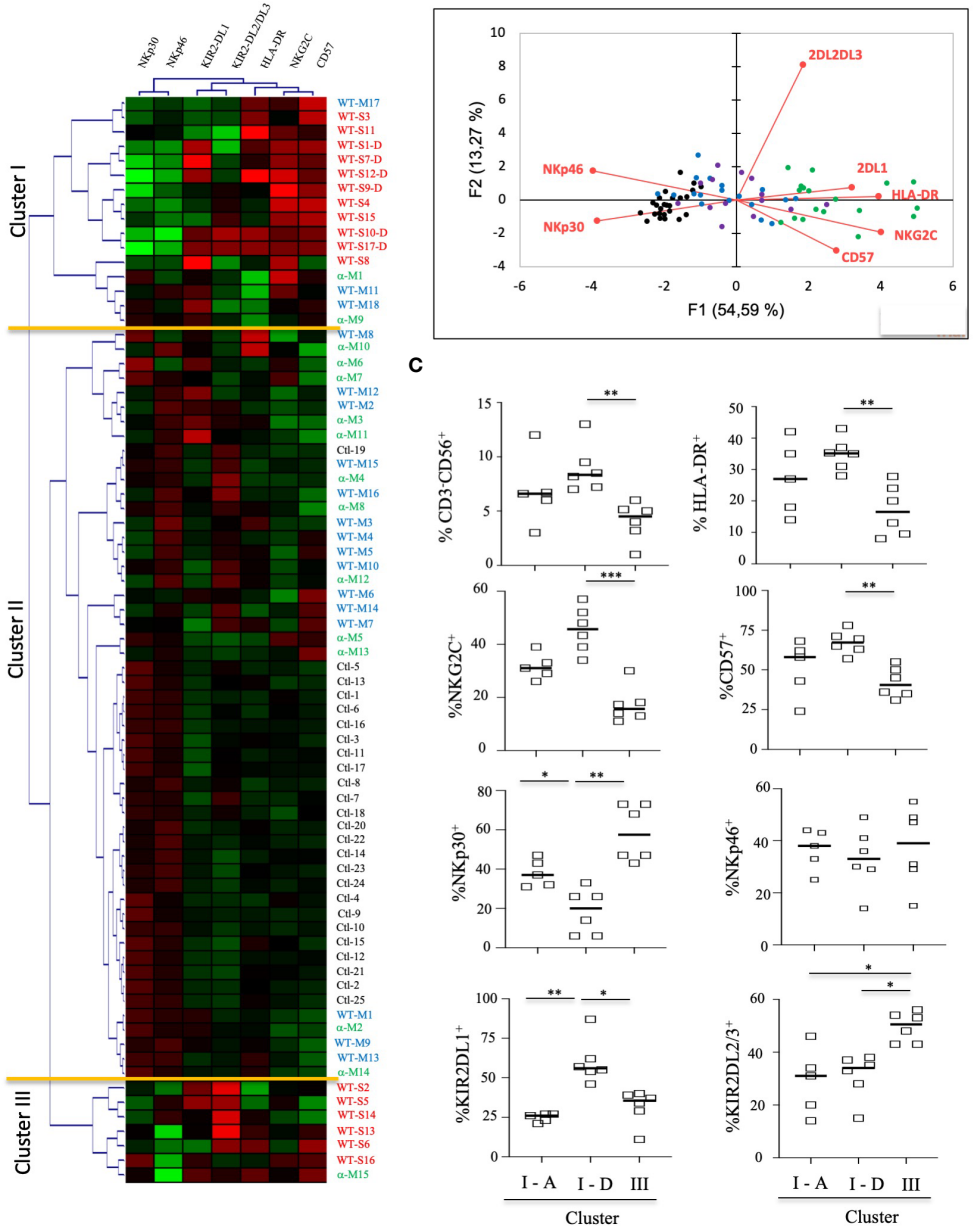
Hierarchical clustering of NK cell markers from COVID-19 patients. **(A)** Clustering analysis of the seven significant cell-surface markers is shown at the first time point (Pt1) for the samples of healthy donors (HD, n=25), patients with severe (WT-S, n=17) or moderate COVID-19 (WT-M, n=18) infected with the WT virus and patients with moderate COVID-19 infected with the Alpha variant (α-M, n=15). Patients with fatal outcomes are indicated by a “D”. Each vertical line is dedicated to a definite NK marker, with the color of each square reflecting the percentage of expression of the corresponding marker in each NK cell sample. The values measured for samples were color displayed and rank ordered considering the healthy donors’ median as a reference: green indicates inferior to the median and red indicates superior to the median. Analysis was performed using the Genesis program (www.genome.tugraz.at). The three different clusters (Cluster I, II and III) are separated by yellow lines. **(B)** Principal component analysis graphically shows the statistical proximity between the different variables that were tested, as well as the distribution of healthy donors (black circles; n=25), patients with severe (green circles, n=17) or moderate COVID-19 (blue circles, n=18) infected with the WT virus and patients with a moderate COVID-19 infected with the Alpha variant (purple circles, n=15). **(C)** Percentage of CD3^-^CD56^+^ and other cell-surface markers among NK cells at the first time point (Pt1) in samples of WT-S patients from clusters I and III. In cluster I, data from surviving (I-A) and deceased (I-D) WT-S patients are presented in two different columns. Black lines represent the median. *p<0.05; **p<0.001; ***p<0.0005.

To better determine the phenotypic characteristics of NK cells in COVID-19 patients regarding their clinical outcome, we performed a hierarchical clustering including the seven markers which were significantly different between infected patients and healthy donors. **Figure 3A** shows three major clusters (I, II and III). Samples from clusters I and III showed highly expressed NKG2C, CD57 and KIRs, while the frequency of NCRs (NKp30 and NKp46) was lower. In contrast, most COVID-19 patients in cluster II segregated in the same way as healthy donors. The 17 patients with severe COVID-19 were only present in clusters I and III, while the six patients with fatal outcomes were only in cluster I. In contrast, only six samples from patients with a moderate form of COVID-19, infected with WT strain (WT-M, n=3) or the Alpha variant (α-M, n=3), segregate in clusters I and III, compared to 15 WT-M and 12 α-M in cluster II (p<0.0001 and p=0.0014, respectively; Fisher’s exact test). Of note, patients from the different clusters present similar characteristics in terms of age, sex, co-morbidity or gravity scores (**Supplementary Table 1**). The principal component analysis (PCA) graphically confirmed that healthy donors and SARS-CoV-2 infected patients have a specific NK cell phenotypic signature. Furthermore, NK cells of patients with severe COVID-19 are more closely associated with HLA-DR, NKG2C, CD57 and KIR2DL1 expression (**Figure 3B**). In order to search for a specific NK cell signature associated with fatality, a re-analysis of samples from clusters I and III was performed. **Figure 3C** shows that patients in clusters I and III were different in terms of NK cell frequency and the expression of HLA-DR, NKG2C, CD57, NKp30 and KIRs markers. Fatality seems to be associated with significantly lower percentage of NKp30 and higher percentages of KIR2DL1 when comparing surviving and deceased patients in cluster I (**Figure 3C**).

Together, these data suggest that most patients with moderate COVID-19 have an NK-cell profile that is similar to that of healthy donors, irrespective of whether they are infected with the WT strain or the Alpha variant. In contrast, patients with severe COVID-19 have a specific signature of adaptive NK cells. However, interestingly, those with fatal outcomes differ from other severe patients by the lower expression of NKp30 associated with a higher frequency of KIR2DL1.

### NK cell functions in COVID-19 patients

**Figure 4:**
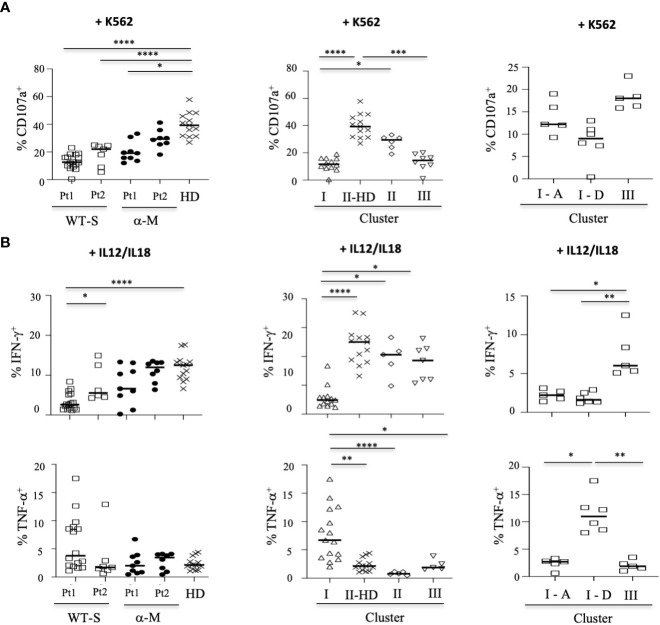
Functional activity of NK cells from COVID-19 patients. **(A)** Degranulation of NK cells measured by the cell-surface expression of CD107a in CD3^-^CD56^+^ NK cells, tested in the presence of the standard K562 target cells (ratio 1:1). Data are taken at two time points (Pt1 and Pt2). **(B)** Intracellular production of IFN-γ or TNF-α in CD3^-^CD56^+^ NK cells after IL-12+IL-18 overnight stimulation (+IL12/IL18). In the left panels, data are shown for the two time points (Pt1 and Pt2) in severe COVID-19^+^ patients infected with WT (WT-S, n=12), or patients with moderate COVID-19 infected by the Alpha variant (α-M, n=9), compared to healthy donors (HD, n=13). In the middle panels, data are shown at the first time point (Pt1) for WT-S and α-M COVID-19 patients from clusters I (n=12), II (n=7) and III (n=8), compared to healthy donors in cluster II (II-HD, n=13). In the right panels, data are shown for WT-S patients from clusters I (n=11) and III (n=5). In cluster I, data from surviving (I-A, n=5) and deceased (I-D, n=6) WT-S patients are presented in two different columns. Black lines represent the median. *p<0.05; **p<0.001; ***p<0.0005, ****p<0.0001.

To further assess the functional properties of NK cells from COVID-19 patients and more specifically those with fatal outcome, we investigated their degranulation capacity. Without target cells, a low level of CD107a expression was observed in NK cells from COVID-19 patients, similar to that observed in healthy donors (**Supplementary Figure 4A**). However, in the presence of the K562 target cells, the degranulation process was significantly decreased in NK cells from patients with severe COVID-19 compared to patients with moderate COVID-19 (p=0.0135) or healthy donors (p<0.0001) (**Figure 4A**). In patients with severe COVID-19, the CD107a frequency remains low irrespective of the timepoint, but returns close to the healthy donor levels for patients with moderate COVID-19 at Pt2 (**Figure 4A**). Of note, the frequency of CD107a was similar in patients with a fatal outcome compared to other patients with severe COVID-19 (compared I-A vs I-D samples in the right panel), suggesting that the low level of degranulation in COVID-19 patients is not associated with fatality.

Next, we measured the ability of NK cells to synthesize and release IFN-γ. In the absence of stimulation, IFN-γ production remains very low, irrespective of the samples tested (**Supplementary Figure 4B**). Following overnight stimulation with IL-12 and IL-18, IFN-γ production was only significantly decreased in patients with severe COVID-19 compared to healthy donors (p<0.0001), but return closed to the normal production at the second time-point (Pt2) (**Figure 4B**). Of note, the production of IFN-γ was similar in CD56^Bright^ and CD56^Dim^ NK-cell subsets, regardless of the group of patients tested (**Supplementary Figure 5**). Interestingly, at Pt1, the intracellular production of IFN-γ was lower in patients of cluster I than in other clusters (**Figure 4B**, middle panel) and more significantly in patients with a fatal outcome (p=0.0256) (**Figure 4B**, right panel).

Concomitantly, the production of TNF-α before stimulation is significantly increased in patients with severe COVID-19 compared to healthy donors (p=0.0001) and patients with moderate disease (p<0.0001) (**Supplementary Figure 4C**, left panel). Furthermore, the production of TNF-a is significantly increased in the CD56^Bright^ NK cells of COVID-19 patients with a severe form (WT-S) at the two time-points (Pt1 and Pt2) compared to healthy donors (**Supplementary Figure 5**). As expected, the increased intracellular production of TNF-α is observed in patients in clusters I and III (**Supplementary Figure 4C**, middle panel) and more significantly in patients with fatal outcomes compared to surviving patients with severe COVID-19 in cluster I (**Supplementary Figure 3C**, right panel). After IL-12 plus IL-18 treatment, a significant increase in TNF-α production is exclusively reported in patients from cluster I (**Figure 4B**, middle panel) and more specifically in those with fatal outcomes (**Figure 4B**, right panel).

**Figure 5:**
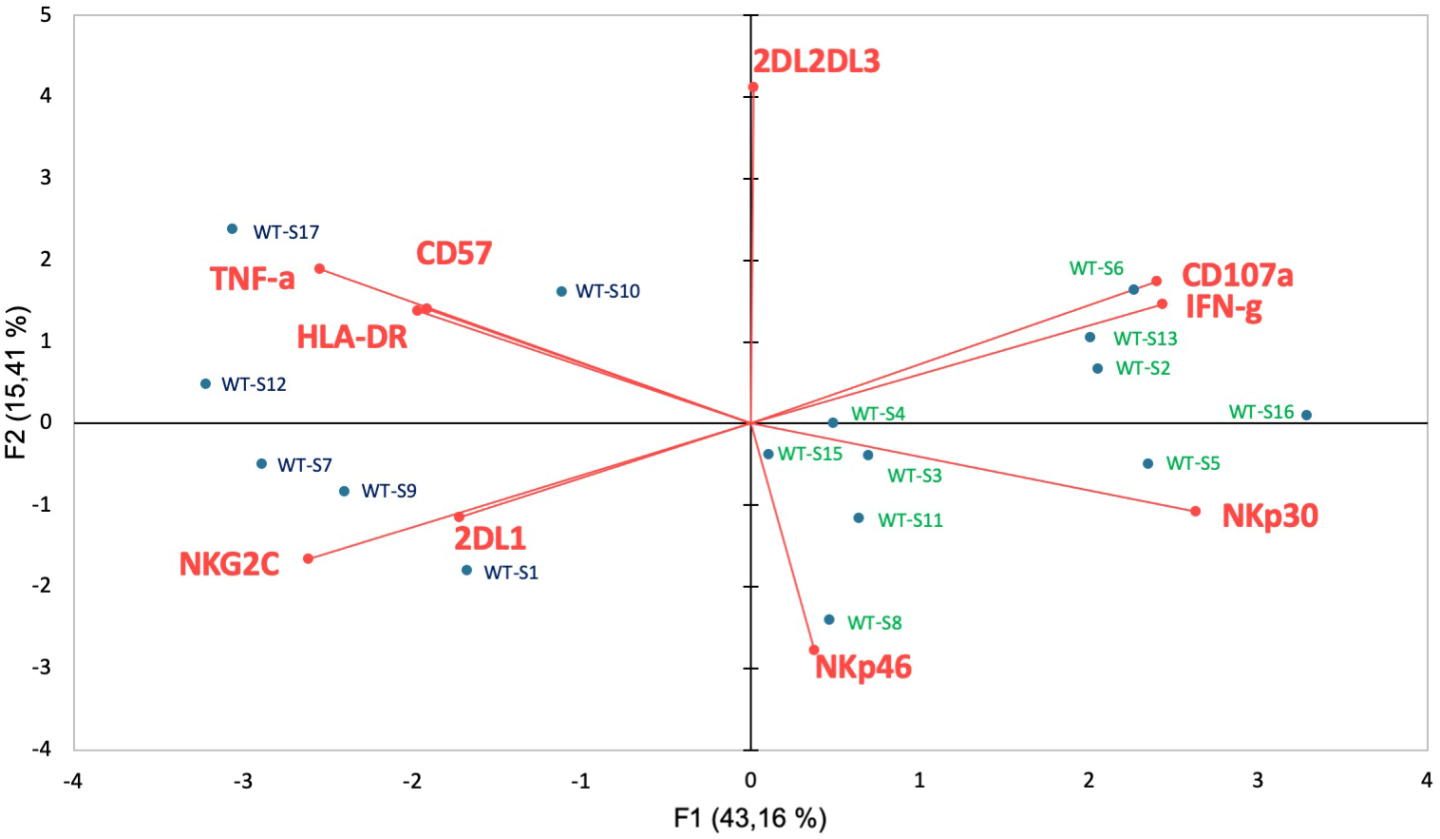
Functional correlation with cell-surface markers associated with fatality. Principal component analysis shows the statistical proximity between the different variables that were tested, as well as the distribution of each patient according to the differential expression of these variables. Blue circles: severely affected patients with a fatal outcome; Green circles: surviving patients with severe COVID-19.

In order to better understand the impact of functional NK cell disturbance in patients with a fatal outcome, we jointly analyzed their phenotypic and functional data. The principal component analysis shows that all severe patients with fatal outcomes cluster together (**Figure 5**) in association with the previously described phenotypic markers HLA-DR, NKG2C, CD57 and KIR2DL1 (**Figures 1B, C**) and TNF-α production. According to these different parameters, this multivariate analysis also shows that severe COVID-19 patients with or without fatal outcomes are two distinct groups.

Overall, these data highlight that NK cells from infected patients and more specifically those with severe COVID-19 are unable to mount a cytotoxic response and produce IFN-γ in the presence of stimuli. In contrast, patients with a fatal outcome have a high capacity to produce TNF-α at high levels, even in the absence of stimuli.

## Discussion

The present study provides novel insights into the role of NK cells in the pathophysiology of patients infected by SARS-CoV-2 and more particularly in patients with fatal outcomes. Although the percentage of NK cells was not modulated in infected patients, the frequency of activated HLA-DR^+^ NK cells is significantly increased in infected patients and more specifically among those with severe COVID-19, in accordance with previous studies (Maucourant et al., 2020; Di Vito et al., 2022).

Based on the fine-tuned balance between inhibitory and activating NK cell receptors, early reports suggested that the increase in the inhibitory NKG2A receptor could contribute to the dysfunctional state of NK cells associated with COVID-19 (Gallardo-Zapata and Maldonado-Bernal, 2022), in contrast to our study and the results from other teams where no modulation of NKG2A was observed (Maucourant et al., 2020). We reported here that the NK cell phenotype of patients with moderate COVID-19 was similar to that of healthy donors, irrespective of the origin of infection (WT vs Alpha variant). In contrast, patients with severe disease present a previously described specific NKG2C^+^ adaptive NK-cell signature (Maucourant et al., 2020). Consistently, genetic variants of the NKG2C/HLA-E axis and in particular the presence of the HLA-E*0101 allele have been previously reported to have a significant impact on the development of severe forms of COVID-19 (Vietzen et al., 2021). In NK cells from patients with a fatal outcome, NKG2C expression is associated with a very significant downmodulation of NKp30 and the overexpression of KIR2DL1, suggesting the presence of a unique NKG2C^high^KIR2DL1^high^NKp30^low^ NK-cell subset associated with fatality. The factors that control this unusual phenotype are currently unknown, but markedly altered NKp30 expression is commonly associated with infection, leading to alterations in dendritic cell (DC)/NK cell crosstalk, as described in HIV infections and HCV (Mavilio et al., 2006; Holder et al., 2013; Lucar et al., 2019). In SARS-CoV-2 infection, it was reported that the deficiency and dysfunction of DCs impaired their ability to induce the proliferation of autologous NK cells, which consequently fail to degranulate and secrete adequate amounts of IFN-γ, in accordance with our functional data and other studies suggesting that NK cells in SARS-CoV-2 infection could exhibit an impairment in effector functions. This is especially seen in patients with severe COVID-19 in terms of cytokine production, degranulation and killing ability against K562 target cells (Bordoni et al., 2020; Kramer et al., 2021; Witkowski et al., 2021; Bi, 2022). The expansion of a “clonal” NKG2C^+^ NK cell subset was also identified as the major reason for perturbations of the KIR repertoire; in human CMV infection, strong NKG2C^+^ NK-cell expansion is dominated by a single inhibitory KIR clone, whereas moderate expansion is more frequently polyclonal (Manser et al., 2019). This “clonal” expansion is largely restricted to HLA-C-specific inhibitory KIRs, whereas the other inhibitory KIRs, such as the HLA-B-specific KIRs, were rarely found to be associated with NKG2C^+^ NK-cell expansion (Lopez-Verges et al., 2011; Béziat et al., 2013). In particular, a strong interaction of KIR2DL1 and HLA-C2 ligands seems to promote the large and stable expansion of adaptive NK cells in human CMV infection (Manser et al., 2019), as observed in this study of COVID-19 patients with fatal outcomes. Possibly, the high-avidity of KIR2DL1 alleles expressed by “clonal” NKG2C^+^ subset impairs the overall control of the viral infection or of these consequences. However, it is currently unclear whether the dominance of a “clonal” NKG2C^high^KIR2DL1^high^NKp30^low^ subset has a regulatory impact on specific T cells controlling virus-specific antibody production, or/and could impair other components of the immune response to consequently explain the clinical outcome. However, it is important to confirm this hypothesis in a larger number of patients with fatal outcomes.

In addition to direct viral damage, uncontrolled inflammation contributes to disease severity in COVID-19, as previously observed in the context of other respiratory viral infections such as those mediated by the respiratory syncytial virus and influenza A virus (Abdul-Careem et al., 2012; Li et al., 2012; Zhou et al., 2013). Here, we observed that TNF-α is strongly produced by NK cells of COVID-19 patients with severe COVID-19 and more significantly in CD56^bright^ NK cells from patients with a fatal outcome. The high production of TNF-α was previously reported in patients infected by HCV in correlation with liver fibrosis and viral load (Nel et al., 2016). In this last viral context, it was shown that hepatic DC acquires the marked ability to stimulate NK cells in a TNF-α-dependent manner in liver fibrosis (Connolly et al., 2009). TNF-α is one of the pro-inflammatory cytokines that is commonly upregulated in acute lung injury (Croft, 2009). In ECMO patients with COVID-19, the level of TNF-α is associated with disease severity and death (Dorgham et al., 2021). Moreover, anti-TNF therapies seem to have had a protective effect on the progression of COVID-19, especially in severe cases of COVID-19, although this needs to be confirmed (Guo et al., 2022). A negative relationship was reported between the degranulation frequency and IFN-γ secretion with respect to high concentrations of pro-inflammatory cytokines and more especially in severe forms of COVID-19 (Gallardo-Zapata and Maldonado-Bernal, 2022). Despite the limited number of COVID-19 patients with fatal outcomes involved in our study, we wondered whether the immunopathogenic process induced by the excessive production of TNF-α mediated by NK cells in SARS-CoV-2 infection could play a role in the fatal outcome.

There are, however, several other limitations to our study, in particular the relatively small number of patients with fatal outcomes in regard to the unexpected data obtained by the clustering study. Furthermore, in an attempt to explain the presence of patients with moderate COVID-19 in clusters I and III, a long-term follow-up could be interesting to search for a possible clinical impact mediated by NK cells in these patients, such as post-acute COVID-19 syndrome (Nalbandian et al., 2021). Finally, we only studied peripheral blood NK cells, whereas it would be interesting to characterize the lung-resident cells to search for the presence of TNF-α-producing NKG2C^high^KIR2DL1^high^NKp30^low^ NK cells. Notably, we have recently reported the presence of NK cells in the pulmonary local broncho-alveolar lavage (BAL) of patients with a severe form that can represent up to 17% of the lymphocytes (Guihot et al., 2022), but their precise phenotyping could not be carried out.

In conclusion, although other studies should be considered to confirm these new data, this study allows us to suggest that a specific subset of TNF-α-producing NK cells could be involved in an immunopathological process leading to fatal forms of COVID-19.

## Data availability statement

The original contributions presented in the study are included in the article/**Supplementary Material**. Further inquiries can be directed to the corresponding author.

## Ethics statement

The studies involving human participants were reviewed and approved by Sorbonne Université. The patients/participants provided their written informed consent to participate in this study.

## Author contributions

NT, VM, AR, KD, CP, OB, PG performed preparation of samples and immune-monitoring of NK cells. EL and AS monitored and analyzed biological data. CS and A-GM performed and analyzed virologic data. AB, JM, RL, J-ML, ZA and C-EL recruited and enrolled patients and monitored clinical data. MM, GG and AG acted as coordinating investigators. VV analyzed the *post-hoc* studies, interpreted the data, and wrote the manuscript. All authors contributed to the article and approved the submitted version.
