# Supplementary material for: Identification of natural killer markers associated with fatal outcome in COVID-19 patients
Source: Front Cell Infect Microbiol. 2023 Jun 5;13:1165756. doi: 10.3389/fcimb.2023.1165756 (PMC10277643; doi:10.3389/fcimb.2023.1165756)
Supplement: Supplementary file 1 [file DataSheet_1.pdf]

Supplementary Material

Identification of natural killer markers associated with fatal outcome in COVID-19 patients

Nadine Tarantino[1], Elena Litvinova[1,2], Assia Samri[1], Cathia Soulié[3], Véronique Morin[1], Alice Rousseau[1], Karim Dorgham[1], Christophe Parizot[2], Olivia Bonduelle[1], Alexandra Beurton[4,5], Makoto Miyara[1,2], Pascale Ghillani[2], Julien Mayaux[4], Raphael Lhote[6], Jean-Marc Lacorte[7,8], Anne-Geneviève Marcelin[3], Zahir Amoura[1,6], Charles-Edouard Luyt[4,6], Guy Gorochov[1,2], Amélie Guihot[1,2], Vincent Vieillard[1*]

*** Correspondence:** Vincent Vieillard: vincent.vieillard@sorbonne-universite.fr

Supplementary Figures and Tables

SUPPLEMENTAL TABLE 1. List of markers used in the study

Marker	Clone	Fluorochrome	Company
CD3	UCHT1	eFluor780	eBioscience
CD56	REA196	PEVio770	Miltenyi Biotec
HLA-DR	I243	AlexaFluor700	BioLegend
CD57	NC1	Pacific Blue	Beckman Coulter
NKG2A (CD159a)	Z199	Phycoerythrin (PE)	Beckman Coulter
NKG2C (CD159c)	134591	Allophycocyanin (APC)	R&D System
NKG2D (CD314)	1D11	BV786	BD Optibuild
NKp30 (CD337)	P30-15	BV421	BD Horizon
NKp46 (CD335)	REA808	PeVio615	Miltenyi Biotec
KIR2DL1 (CD158a)	HP-3E4	Fluorescein isothio-cyanate (FITC)	BD Pharmingen
KIR2DL2/DL3 (CD158b/b2,j)	GL183	Phycoerythrin (PE)	Beckman Coulter
KIR3DL1 (CD158e1)	DX9	AlexaFluor700	Biolegend
ILT2 (CD85j)	GHI/75	Allophycocyanin (APC)	Miltenyi Biotec
CD107a	H4A3	Fluorescein isothio-cyanate (FITC)	BD Pharmingen
IFN-γ	B27	AlexaFluor700	BD Pharmingen
TNF-α	Mab11	eFluor450	eBioscience
Viability		eFluor506	eBioscience

SUPPLEMENTAL TABLE 2. Demographic and clinical characteristics of COVID-19$^+$ patients divided into the three clusters

	Cluster I	Cluster II	Cluster III	P Cluster I vs II
Number; n	16	27	7	0.0428
Sex; n of Female (%)	4 (25.0)	10 (37.0)	2 (28.6)	ns
Composition*				
WT-S; n (%)	11 (64.7)	0 (0)	6 (35.3)	0.0001
WT-M; n (%)	3 (16.7)	15 (83.3)	0 (0)	0.0002
a-M; n (%)	2 (13.3)	12 (66.7)	1 (6.6)	0.0007
Mean age in Yrs (ranged)	66 (36-83)	57 (27-94)	50 (23-67)	ns
Co-morbidities				
Obesity; n (%)	8 (50.0)	9 (33.3)	5 (71.4)	ns
Diabetes (type 1 and 2); n (%)	6 (37.5)	5 (18.5)	3 (42.9)	ns
Chronic cardiac disease; n (%)	2 (12.5)	4 (14.8)	2 (28.6)	ns
Hypertension; n (%)	7 (43.8)	17 (63.0)	4 (57.1)	ns
Gravity scores				
IGS II at baseline; mean±SD	39.6±17.9	28.3±1.9	37.3±14.2	ns
SOFA; mean±SD	12.4±6.3	ND	9.5±3.5	ND
Respiratory support (ECMO); n (%)	4 (25.0)	0 (0)	2 (28.6)	0.0147
ARDS; n (%)	4 (25.0)	1 (3.7)	2 (28.6)	ns
Mortality at hospital; n (%)	6 (37.5)	0 (0)	0 (0)	0.0013

*the composition is determined according to the total number of patients in the group, including patients with moderate disease infected with the original strain (WT-M) or the Alpha variant (□-M), and patients with severe disease infected with the original strain (WT-S). IGS II: *Simplify Gravity Index*; SOFA: Sequential Organ Failure Assessment; ECMO: Extracorporeal Membrane Oxygenation; *ARDS*: *Acute respiratory distress syndrome*.

SUPPLEMENTAL TABLE 3. Individual characteristics of COVID-19^{+} patients admitted in ICU with fatal outcome

	Age in Yrs	Sex M/F	Co-morbidity				Pulmonary infection	ECMO/ ARDS	IGSII baseline	Time to fatality in Days*
			Obsesity	Diabetes	Cardiac disease	Hypertension				
WT-S1	51	M	1	0	0	1	Aspergillosis infection	1/1	66	20
WT-S7	69	M	0	0	0	1	SOFA	0/1	14	10
WT-S9	58	M	1	0	0	0	Bacterial pneumonia, ATB	1/1	31	6
WT-S10	62	F	1	1	0	1	Aspergillosis infection & SOFA	0/0	50	24
WT-S12	55	M	1	0	0	0	SOFA	1/1	54	23
WT-S17	69	M	1	1	0	1	Bacterial pneumonia, ATB	0/0	41	15

M/F: Male/Female; ICU: Intensive care unit; ECMO: extracorporeal membrane oxygenation; ARDS Acure respiratory distress syndrome; IGSII: Simplify gravity index; SOFA, Sepsis-related Organ Failure Assessment; ATB: Antibiotic therapy. *Time to fatality in Days: length of-stay in ICU before fatal outcome

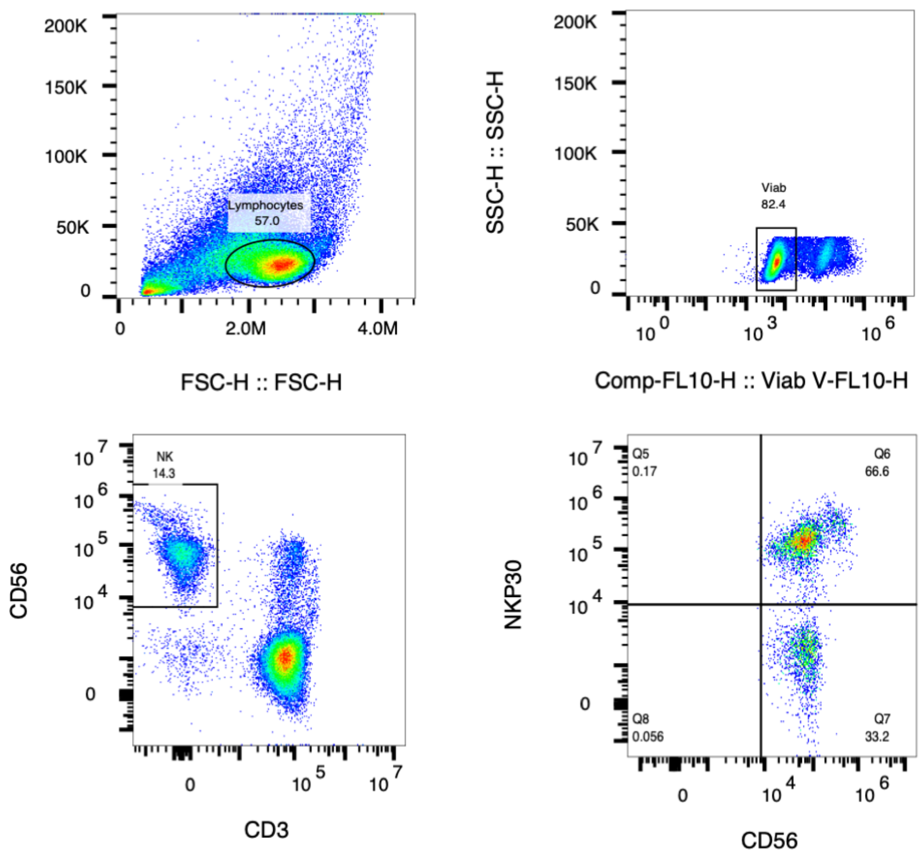

SUPPLEMENTAL FIGURE 1.

Gating strategy for FACS analysis from a representative COVID-19 patient. From the lymphocyte gate, obtained by the size (SSC) and structure FSC); The $CD3^-CD56^+$ NK cells were identified within the alive lymphocytic population. The different makers, like NKp30, are then independently analyzed on the NK cell gate.

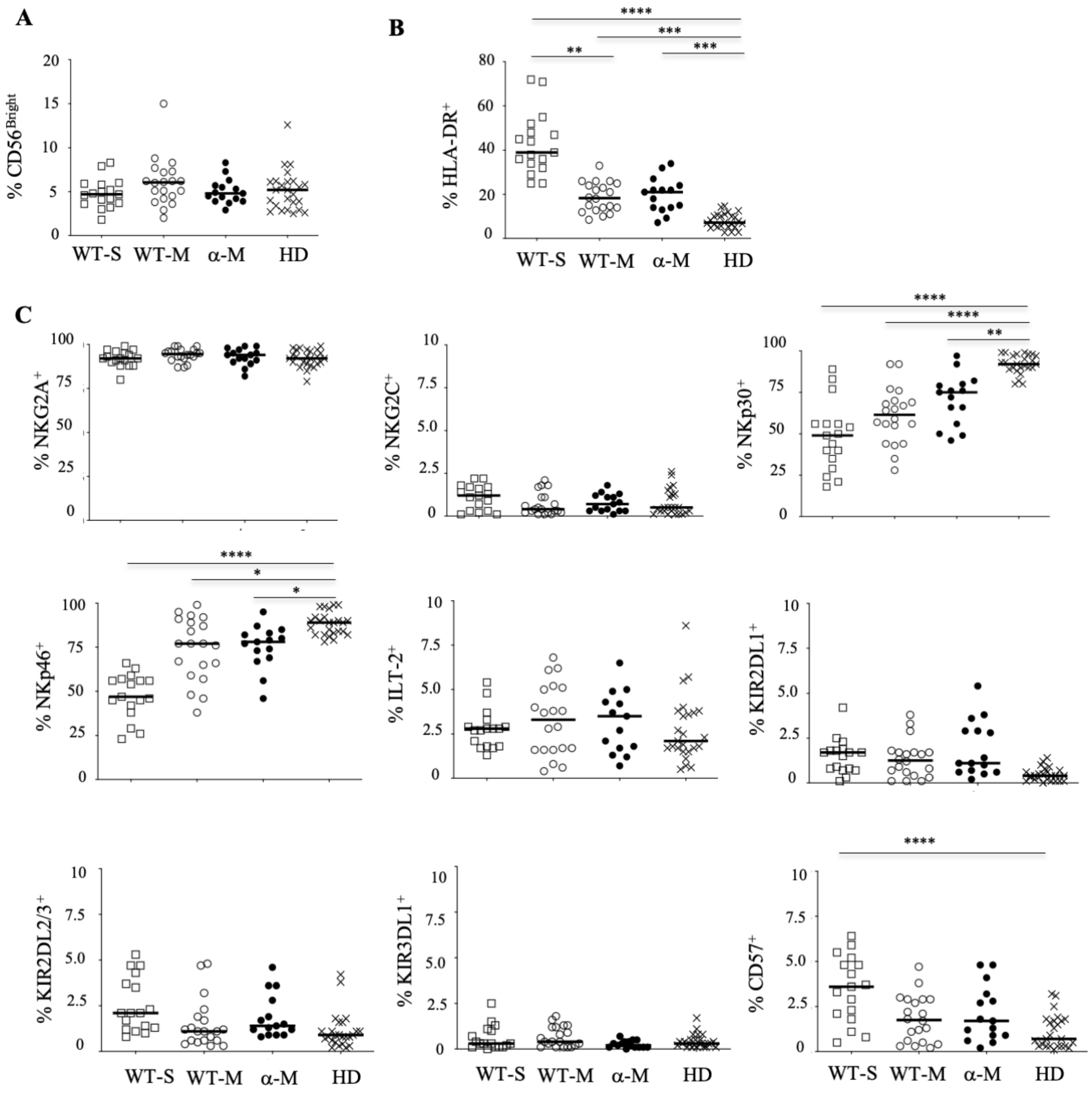

SUPPLEMENTAL FIGURE 2.

Analysis of $CD56^{Bright}$ NK-cell subset. **(A)** Frequency of $CD56^{Bright}$ NK within the $CD3^{-}CD56^{+}$ NK cell gate. **(B)** Percentage of cells expressing cell-surface markers among $CD56^{bright}$ NK cells. Data are shown for healthy donors (HD, n=25), and at the first time point (Pt1) for patients with severe (WT-S, n=17) or moderate COVID-19 (WT-M, n=18) infected by WT virus, or patients with moderate COVID-19 infected by Alpha variant (α-M, n=15). Black lines represent the median. *$p<0.05$; **$p<0.001$; ***$p<0.0005$, ****$p<0.0001$.

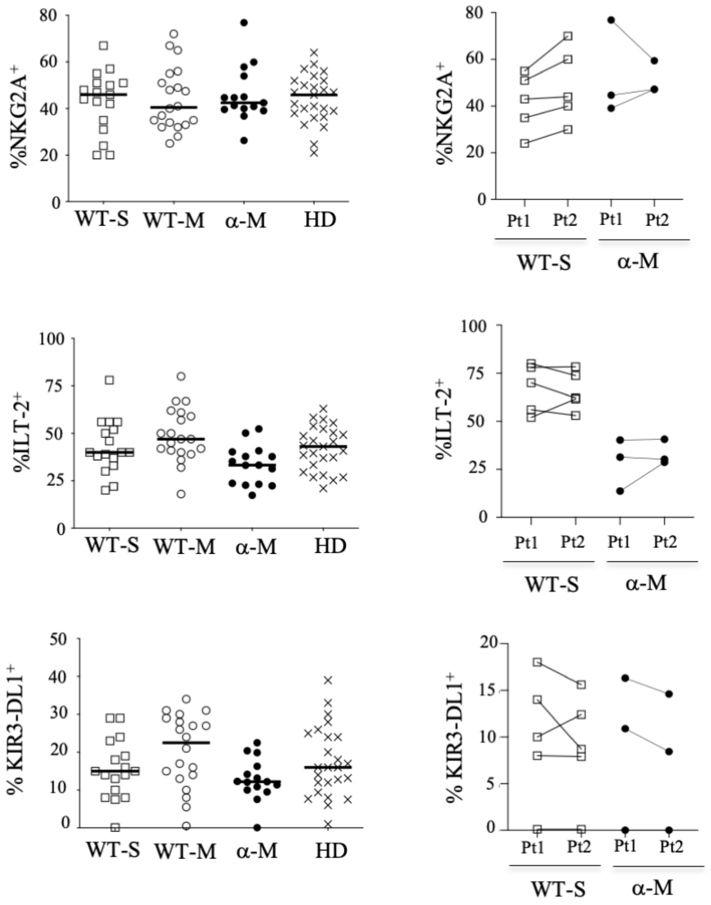

SUPPLEMENTAL FIGURE 3.

Frequency of NKG2A, ILT-2 and KIR3-DL1 on CD3$^-$CD56$^+$ NK cells. Data are shown for healthy donors (HD, n=25), and patients at the first time point (Pt1) with severe (WT-S, n=17) or moderate COVID-19 (WT-M, n=18) infected by WT virus, as well as patients with moderate or COVID-19 infected by Alpha variant (α-M, n=15). Black lines represent the median. For some samples, data are done at two time-points (Pt1 and Pt2, right panels).

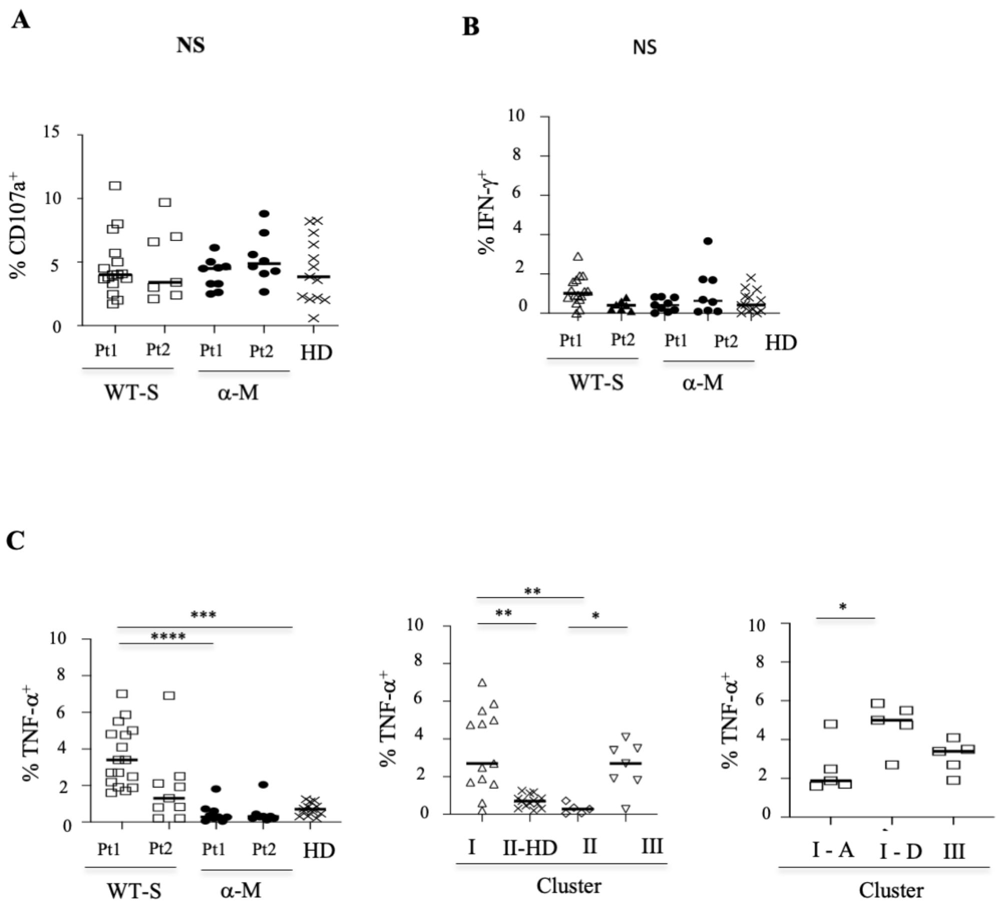

SUPPLEMENTAL FIGURE 4

Functional activity of NK cells from COVID-19 patients in non-stimulated samples. **(A)** Degranulation of NK cells measured by cell-surface expression of CD107a on $CD3^-CD56^+$ NK cells (NS). **(B)** Intracellular production of IFN-γ in $CD3^-CD56^+$ NK cells (NS). **(C)** Intracellular production of TNF-α in $CD3^-CD56^+$ NK cells (NS). In the left panel, data are done for healthy donors (HD, n=13), and at two-time points (Pt1 and Pt2) for patients with severe COVID-19 infected by WT virus (WT-S, n=12), or with moderate COVID-19 infected by the Alpha variant (α-M, n=9). In the middle panel, data are done at the first time point (Pt1) for WT-S and α-M COVID-19 patients from clusters I (n=12), II (n=7) and III (n=8), compared to healthy donors (II-HD, n=13). Clusters are defined in Figure 3A. In the right panel, data are shown at the first time point (Pt1) for WT-S patients from clusters I (n=11) and III (n=5). Data from alive (I-A, n=5) and deceased (I-D, n=6) WT-S patients of the cluster I are presented in two different groups. Black lines represent the median. *p<0.05; **p<0.001; ***p<0.0005, ****p<0.0001.

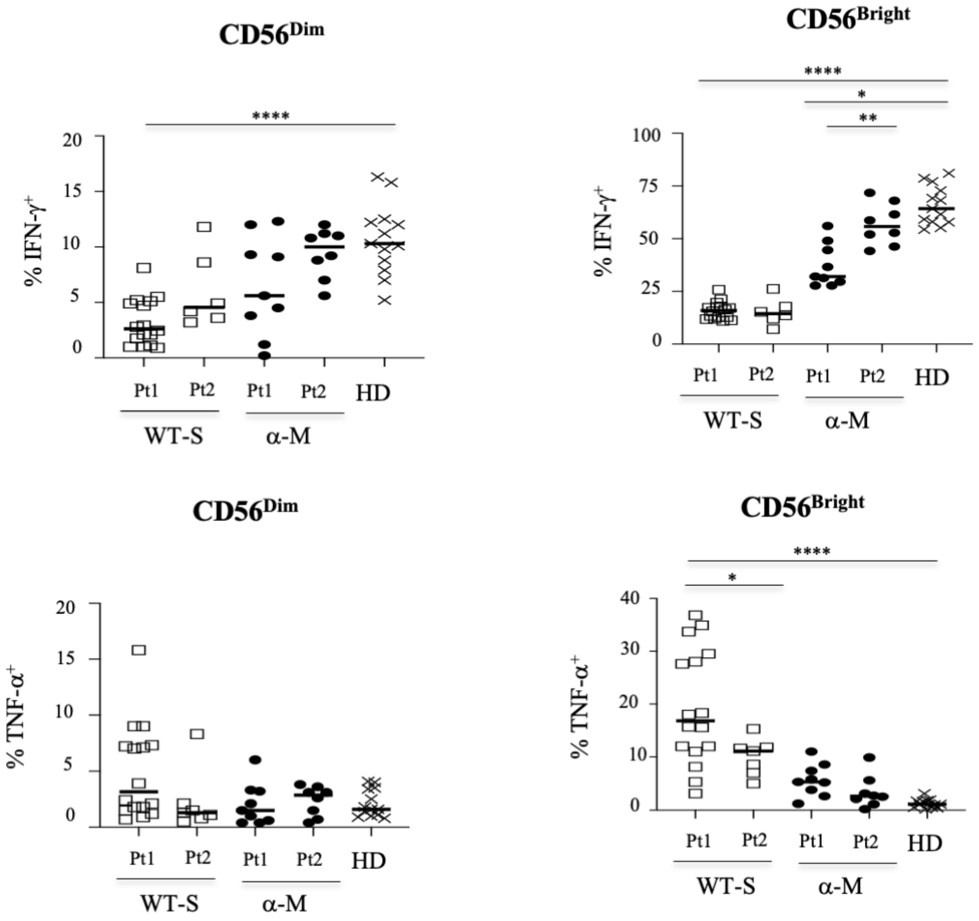

SUPPLEMENTAL FIGURE 5

Intracellular cytokine production of NK cells from COVID-19 patients in IL-12/IL-18 stimulated samples. Data done for healthy donors (HD, n=13), and at two-time points (Pt1 and Pt2) for patients with for severe COVID-19 infected by WT virus (WT-S, n=12), or with moderate COVID-19 infected by the Alpha variant (α-M, n=9). Black lines represent the median. *p<0.05; **p<0.001; ***p<0.0005, ****p<0.0001.
